# Unsaturated fatty acid salts remove biofilms on dentures

**DOI:** 10.1038/s41598-021-92044-y

**Published:** 2021-06-15

**Authors:** Teruyuki Hara, Atsunori Sonoi, Takuya Handa, Masayuki Okamoto, Eri Kaneko, Reiko Ikeda, Taichi Habe, Hidetake Fujinaka, Shigeto Inoue, Tetsuo Ichikawa

**Affiliations:** 1 Analytical Science Research Laboratories, Kao Corporation, 1334 Minato, Wakayama-shi, Wakayama 640-8580 Japan; 2 Personal Health Care Products Research Laboratories, Kao Corporation, 2-1-3 Bunka, Sumida-ku, Tokyo 131-8501 Japan; 3 Department of Prosthodontics and Oral Rehabilitation, Graduate School of Biomedical Sciences, Tokushima University, 3-18-15 Kuramoto, Tokushima, 770-8504 Japan

**Keywords:** Prosthetic dentistry, Dentistry, Dental public health, Oral hygiene, Microscopy, Mass spectrometry, Biofilms

## Abstract

Candidiasis-causing *Candida* sp. forms biofilms with various oral bacteria in the dentures of the elderly, making it harder to kill and remove the microorganism due to the extracellular polymeric substances. We found that biofilms on dentures can effectively be removed by immersion in an unsaturated fatty acid salt solution. Using optical coherence tomography to observe the progression of biofilm removal by the fatty acid salt solution, we were able to determine that the removal was accompanied by the production of gaps at the interface between the biofilm and denture resin. Furthermore, microstructural electron microscopy observations and time-of-flight secondary ion mass spectrometry elucidated the site of action, revealing that localization of the fatty acid salt at the biofilm/denture-resin interface is an important factor.

## Introduction

Candidiasis is a typical opportunistic infection caused by abnormalities in the local or systemic defense mechanisms of the patient^1,2^; it is common and tends to recur and become intractable in the elderly due to their weakened immunity^3^.

Symptoms such as redness and pain in the oral mucosa and taste disorders^4^ are associated with oral candidiasis, which is caused mainly by *Candida albicans*^1,2^. In the oral cavity, *Candida* sp. are also reportedly involved in periodontal disease, denture stomatitis^5^, and median rhomboid glossitis^6^. These species have also been implicated as reservoirs for *Helicobacter pylori*^7,8^, which causes gastric ulcers, and is also a cause of systemic diseases, such as aspiration pneumonia, bacterial endocarditis, as well as gastrointestinal and respiratory infections^9^.

*Candida* spp. manifest in the oral cavities of the elderly with reduced immunity due to medication or systemic disease but are also frequently detected on the surfaces of dentures where they form biofilms with indigenous oral bacteria, such as *Streptococcus* sp.^10^.

Chemical antiseptics are commonly used to treat oral microorganisms; however, they are limited in their efficacy once biofilms are formed because the extracellular polymeric substances (EPS) produced by the bacteria act as a barrier that prevents the antiseptic from penetrating into the cells^11^. Even if the antiseptic penetrates the EPS and kills the bacteria in the biofilm, components of the biofilm, such as its lipopolysaccharides, lipoproteins, and bacterial DNA, can themselves be toxic^12^. The EPS is also known to contribute to the growth of the biofilm^13,14^. The thorough removal of biofilms on dentures is therefore important because it protects the elderly from a variety of diseases and improves their quality of life.

Meanwhile, fatty acids and fatty acid salts are known to exhibit antibacterial effects on oral bacteria^15,16^. The antimicrobial properties of fatty acid salts against *S. mutans* tend to be higher in medium-chain fatty acid salts of C10 to C14 and unsaturated fatty acid salts of C18 than in short-chain fatty acid salts of C8 or less. The mechanism behind antibacterial activity of fatty acid salts is still unclear. Short-chain fatty acids are ineffective probably because their alkyl chain length is too short to adsorb onto the cell surface. Hypotheses regarding unsaturated fatty acid salts include the incorporation of the cis-bonds of unsaturated fatty acids into the membrane^17^, their inhibition of fatty acid biosynthesis^18^. Oral bacteria in a biofilm are also known to produce fatty acids that act as endogenous antibacterial agents, inhibiting the growth of other species in the biofilm^19^ and suppressing plaque formation^20^. However, there are very few reports on the effects of exogenous fatty acid salts on biofilms formed on denture resins. Interestingly, in this study we show for the first time that simply immersing a denture biofilm model in a fatty acid salt solution leads to the detachment of the biofilm from the surface of poly(methyl methacrylate) (PMMA)^21^, a common denture material. The protocol developed in this study has the potential to protect older people from a wide range of diseases by thoroughly removing the biofilms formed on their dentures. Furthermore, since the biofilm-removal mechanism is not well understood, we examined in detail how the fatty acid salt solution removes the biofilm on a denture resin by identifying the main site of action.

Observing the dynamic removal of a biofilm in situ can shed light on the mechanism of detachment of the biofilm from the substrate using unsaturated fatty acid salt treatment; however, observational methods capable of achieving this are limited. For example, electron microscopy requires pretreatment, such as staining and dehydration fixation, which can alter the original configuration^22^. In addition, these observational techniques require very high vacuum environments, which are inconducive for studying dynamic processes. Conversely, optical coherence tomography (OCT) enables acquisition of tomographic images on the micrometer scale in a non-destructive manner so far as the object is transparent to light. This technique has been effectively used to observe morphological changes in biofilms in the water quality protection field^23^. Therefore, we used OCT to observe the three-dimensional structural changes undergone by biofilms, including the ones at the biofilm/PMMA-substrate interface. Time-of-flight secondary ion mass spectrometry (ToF–SIMS) can directly detect target molecules and is being increasingly used to characterize biological materials^24^. Furthermore, organic materials can be depth-profiled by surface sputtering with a gas cluster ion beam (GCIB)^25^. In this study, we used these as well as microstructural analysis techniques, such as electron microscopy, to analyze the site of action of the fatty acid salt on the biofilm for investigating the removal mechanism of biofilm. Oleate, linoleate, and linolenate were selected as the medium-chain and unsaturated fatty acid salts, and laurate was selected as the medium-chain saturated fatty acid salt as they are resolvable in water at room temperature.

## Results

### Biofilm formation on denture resin and its removal by potassium oleate

**Figure 1:**
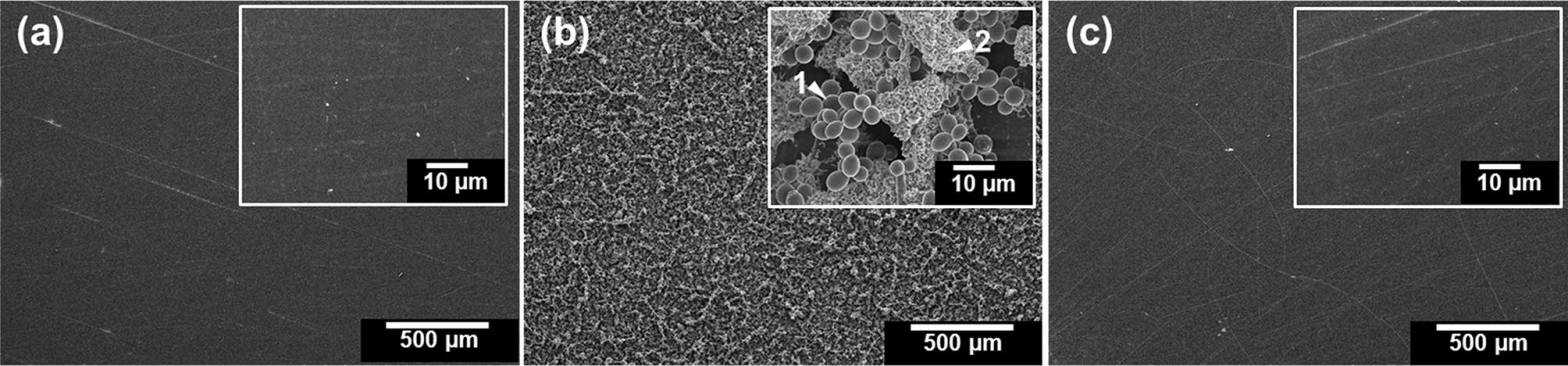
SEM images of PMMA-substrate surfaces at 50 × magnification (Insets: 1000 × magnification). (**a**) Untreated PMMA substrate. (**b**) Co-cultured *C. albicans* and *S. mutans* biofilm formed on the PMMA substrate. Arrow 1: *C. albicans*; arrow 2: *S. mutans*. (**c**) Biofilm-overgrown PMMA substrate after immersion in 30 mM oleate solution for 10 min. ImageJ v1.58 (https://imagej.nih.gov/ij/) and Microsoft PowerPoint have been used to prepare this figure.

Here we describe the structure of the biofilm formed on the denture resin and the process of removal using a potassium oleate (an unsaturated fatty acid salt) solution. Figure 1b shows a scanning electron microscopy (SEM) image of a biofilm composed of *C. albicans* and *S. mutans* which was prepared on a PMMA substrate as a denture resin model. In addition to *C. albicans* and *S. mutans* cells, aggregates of bacterial cells that are partially covered by the EPS produced by *S. mutans* are visible. The biofilm was effectively removed when the PMMA substrate on which the biofilm had formed was immersed for 10 min in a 30 mM potassium oleate solution (Fig. 1c); this was confirmed by comparison with the SEM image of the original PMMA substrate (Fig. 1a). Note that the concentration of 30 mM was determined from prior studies on optimum concentration required for effective removal of the biofilm.

### Changes in the biofilm on a PMMA substrate after treatment with fatty acid salt

**Figure 2:**
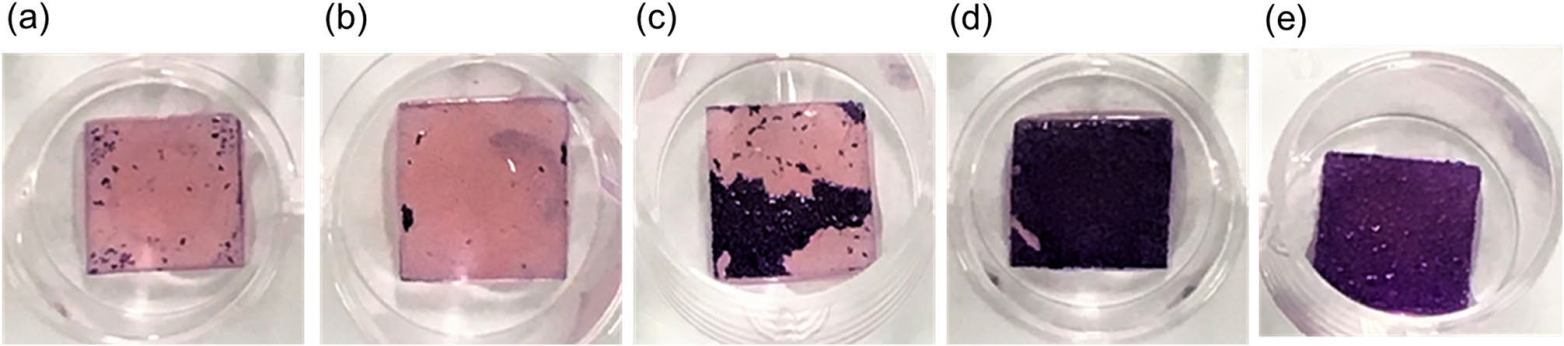
Crystal violet-stained PMMA substrates overgrown with co-cultured *C. albicans* and *S. mutans* biofilms immersed in various 30 mM fatty acid salt solutions for 10 min: (**a**) oleate, (**b**) linoleate, (**c**) linolenate, (**d**) laurate, and (**e**) deionized water. ImageJ v1.58 (https://imagej.nih.gov/ij/) and Microsoft PowerPoint have been used to prepare this figure.

To characterize the effects of various fatty acid salts on the biofilm, we selected oleate (18:1), linoleate (18:2), and linolenate (18:3) salts as the unsaturated fatty acid salts and sodium laurate as the saturated fatty acid salt. Biofilms formed by the co-cultivation of *C. albicans* and *S. mutans* on the PMMA substrates were immersed in the solutions of these fatty acid salts. The biofilm removal was observed (Fig. 2), and the amount of biofilm that remained was determined via crystal violet assay.

**Figure 3:**
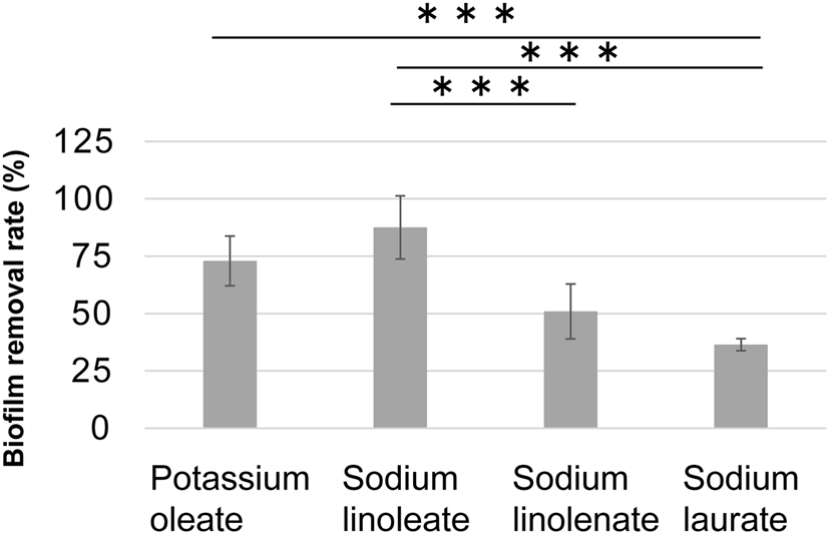
Co-cultured *C. albicans* and *S. mutans* biofilm-removal rates after immersion in various 30 mM fatty acid salt solutions for 10 min. Biofilm removal was quantified via crystal violet assay. The results are shown as means ± s.e.m. (n = 9–13). ***:*p* < 0.005 for the comparison among groups. The asterisks indicate statistically significant differences between the groups. Adobe Illustrator (CS) have been used to prepare this figure.

Immersion treatment with solutions of the abovementioned unsaturated fatty acid salts, especially oleate and linoleate, resulted in high rates of biofilm removal (Fig. 3). In addition, we found that the unsaturated fatty acid salt treatment resulted in the detachment of biofilm from the surface of PMMA substrate (Fig. 2a–c). In contrast, the treatment with the saturated fatty acid salt did not result in film detachment (Fig. 2d), treatment with deionized water (the control) was also similar (Fig. 2e).

### Observation of the biofilm-removal process by optical coherence tomography (OCT)

**Figure 4:**
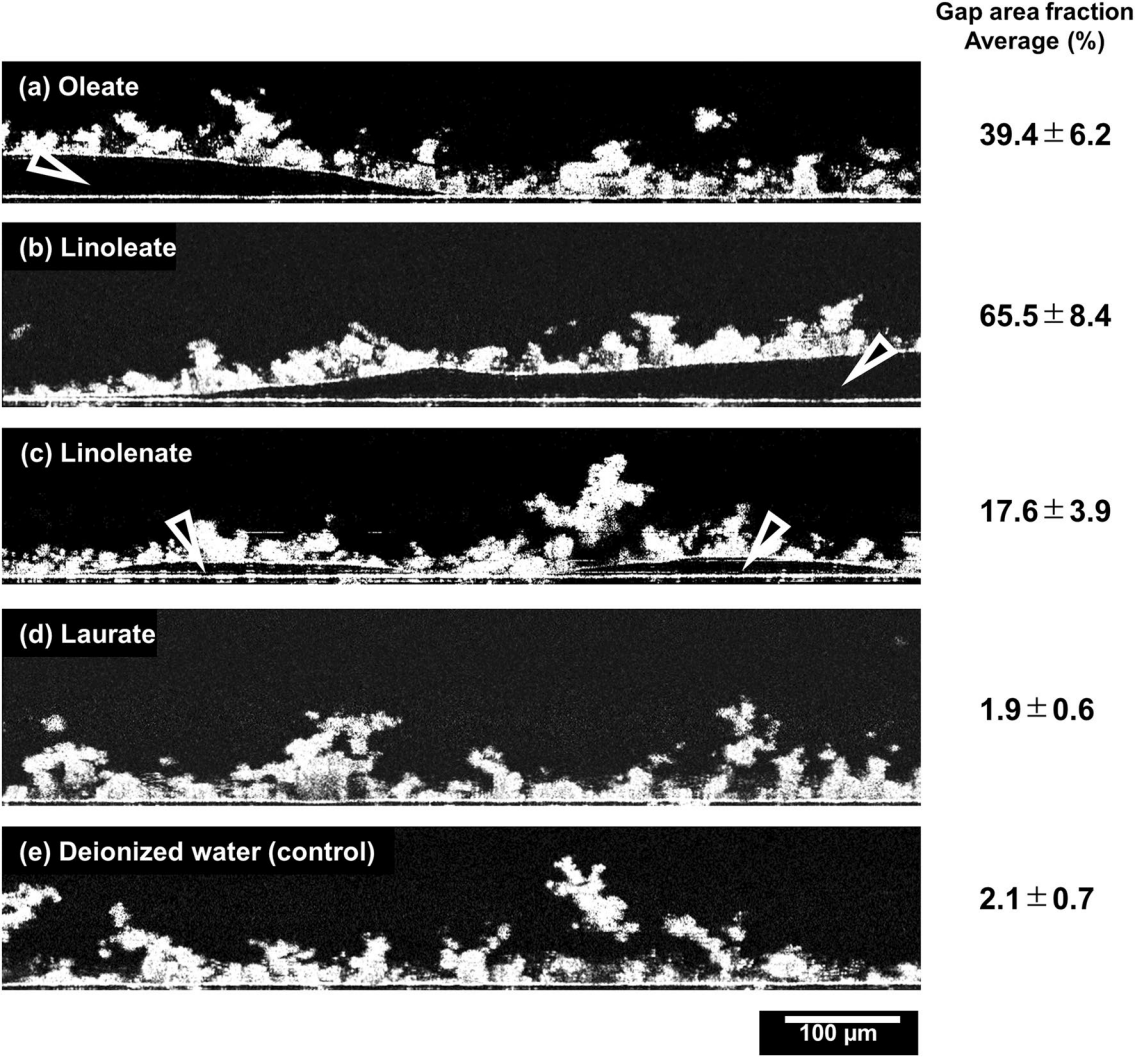
Optical coherence tomography (OCT) images of co-cultured *C. albicans* and *S. mutans* biofilms after immersion in deionized water and various 30 mM fatty acid salt solutions for 10 min. The biofilms are depicted in white and water in black. The arrows in panels (**a**–**c**) indicate the gaps produced at the biofilm/PMMA-substrate interface. (**a**) Oleate, (**b**) linoleate, (**c**) linolenate, (**d**) laurate, and (**e**) deionized water. Area fraction of gaps at biofilm/PMMA-substrate are inset. ImageJ v1.58 (https://imagej.nih.gov/ij/) and Microsoft PowerPoint have been used to prepare this figure.

We used OCT to observe the three-dimensional structural changes undergone by biofilms, including the ones at the biofilm/PMMA-substrate interface. The PMMA substrate on which the biofilm had formed was inserted into the in situ measurement cell and observed by OCT. The biofilm was found to be denser closer to the interface with the PMMA substrate, which is consistent with the previous reports^26^. Then, we examined the changes in the biofilm after immersion in each fatty acid salt solution. No changes in the biofilm/PMMA-substrate interface were observed when the biofilm was treated with deionized water (the control) and laurate solution (Fig. 4d,e). On the other hand, treatment with the oleate, linoleate, and linolenate solutions, which are capable of detaching the biofilm, resulted in the formation of multiple gaps at the biofilm/PMMA-substrate interface (Fig. 4a–c). The area fraction of the gaps at the biofilm/PMMA-substrate interface (x–y plane) was calculated, and the results are shown in Fig. 4 (details are included in supporting information Fig. S1). These results quantitatively show the association between the occurrence of gaps and biofilm removal.

### Site-of-action analysis by precise depth profiling

Traditionally, the removal of a biofilm by chemical treatment is believed to progress gradually from the surface layer of the biofilm^27^. However, our OCT observations of detachment at the biofilm/PMMA-substrate interface strongly suggest that the fatty acid salts act directly at the biofilm/PMMA-substrate interface. We examined the interface to verify that the site where the fatty acid salt acts on the biofilm is the interface.

**Figure 5:**
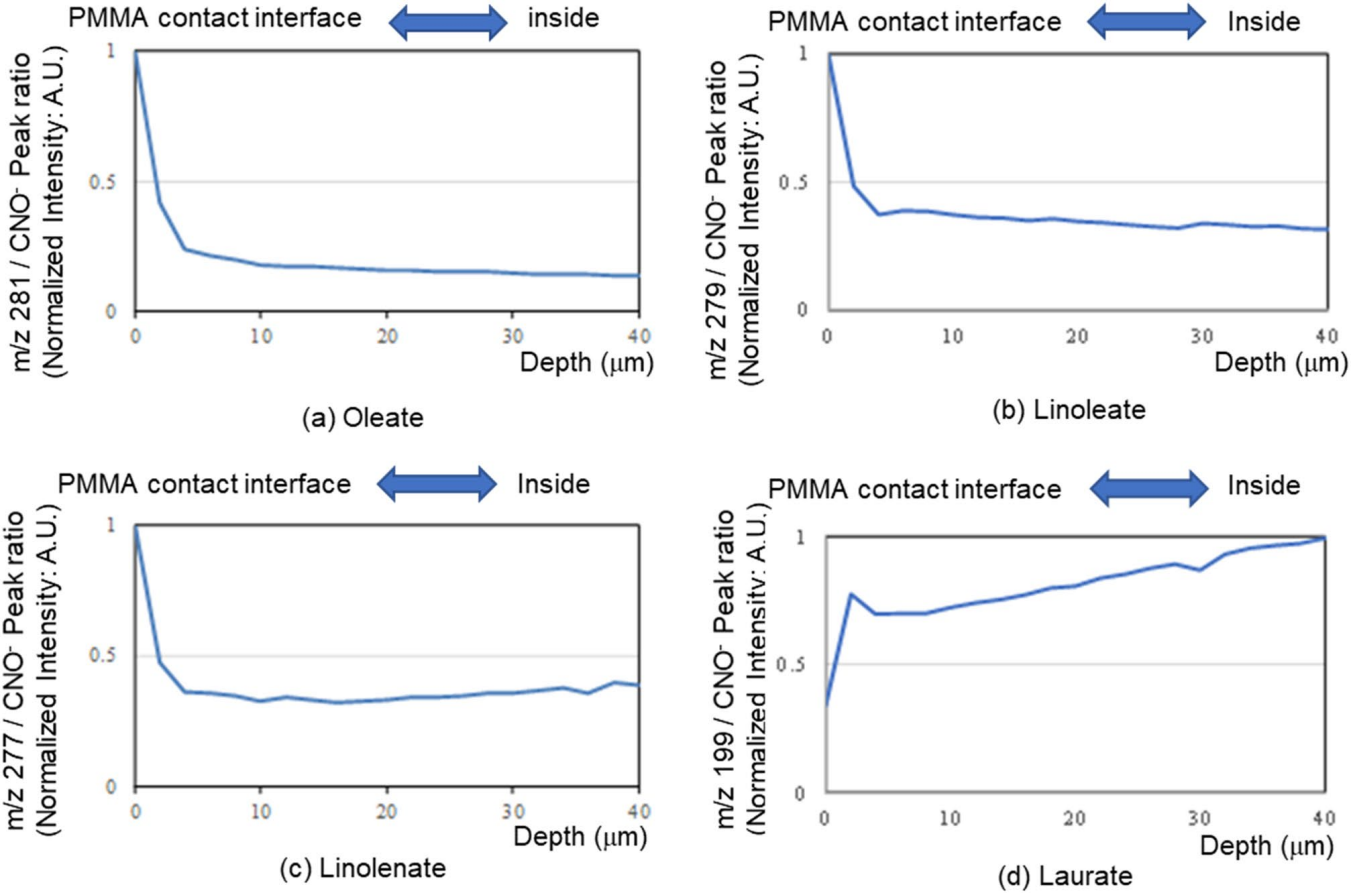
Depth profiles of various fatty acids determined from the side of the interface in contact with PMMA substrate. Microsoft PowerPoint have been used to prepare this figure.

First, oleate was selected as a typical fatty acid salt capable of detaching the biofilm. Biofilms treated with potassium oleate solution were freeze-dried^28^ for ToF–SIMS. By GCIB sputtering the side of the biofilm in contact with the PMMA substrate, we successfully depth-profiled oleate from the biofilm/PMMA-substrate interface by monitoring the negative molecular ion derived from oleic acid (at *m/z* 281) (Fig. 5a), which revealed that oleate was localized at the biofilm/PMMA-substrate interface. Similar results were obtained for the linoleate and linolenate salts, which also exhibited abilities to detach the biofilm (Fig. 5b,c). In contrast, laurate, which does not remove the biofilm, was not localized at the biofilm/PMMA-substrate interface (Fig. 5d).

### Microstructural characteristics of the biofilms

**Figure 6:**
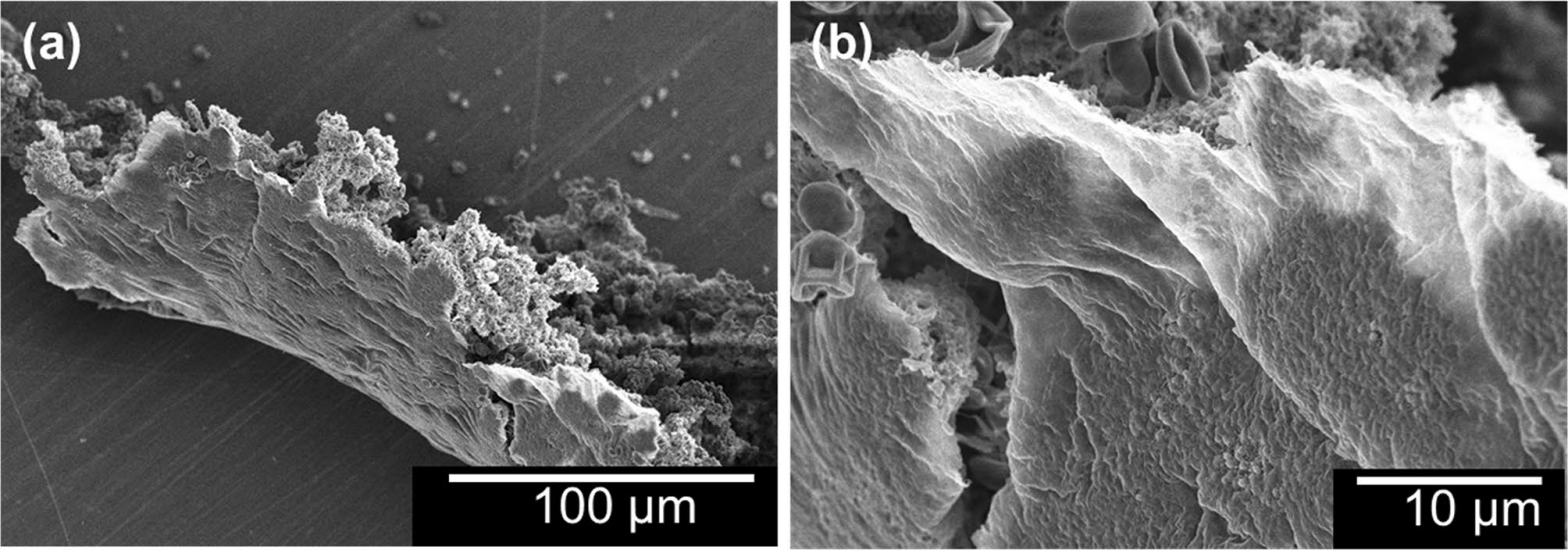
SEM images of a residual fragment of a co-cultured *C. albicans* and *S. mutans* biofilm after immersion in 30 mM oleate for 10 min: (**a**) 500 × magnification and (**b**) 3000 × magnification. ImageJ v1.58 (https://imagej.nih.gov/ij/) and Microsoft Powerpoint have been used to prepare this figure.

**Figure 7:**
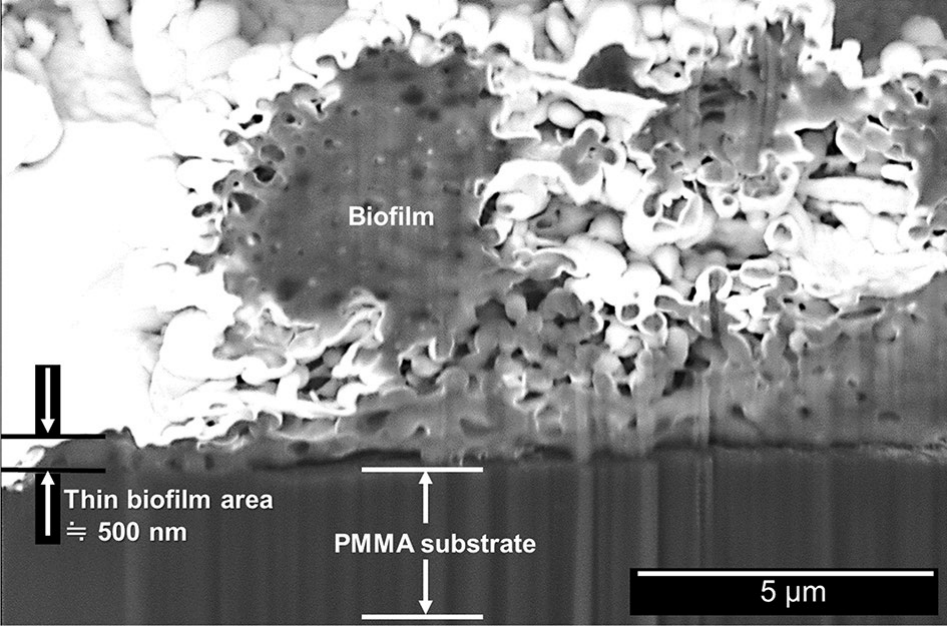
Backscattered-electron SEM image of a cross-section created by FIB cutting. The high-intensity area on the biofilm surface is the platinum protective film used during FIB cutting. ImageJ v1.58 (https://imagej.nih.gov/ij/) and Microsoft PowerPoint have been used to prepare this figure.

Figure 6 shows SEM images of a residual fragment of a co-cultured *C. albicans* and *S. mutans* biofilm after immersion in 30 mM oleate for 10 min. Figure 6a shows the “mountains and valleys” structure of biofilm on the macroscale, and this image is a good example of the biofilm peeled off as a film. Mesh-like membrane structures were observed in a high magnification image (Fig. 6b). To understand the microstructure of the “mesh-like membrane,” we observed backscattered-electron SEM image of a cross-section created by focused ion beam (FIB) cutting, and we found that the “thin-biofilm region” existed on the PMMA substrate (Fig. 7).

## Discussion

We found that a biofilm can be detached from the surface of a PMMA substrate by immersion in a solution of an unsaturated fatty acid salt (oleate, linoleate, or linolenate). This observation is noteworthy because only 10 min of immersion in the solution and slight agitation was required without any brushing or use of other physical forces. SEM of the treated PMMA substrate showed that very little biofilm remained, which indicates that the biofilm was effectively removed from the PMMA-substrate surface and that immersion treatment with an unsaturated fatty acid salt solution is a highly effective and easy-to-use technique for biofilm removal. Notably, the biofilm peeled off as a film (Fig. 3a–c), which suggests that its removal proceeds via a mechanism that is significantly different from the approaches that remove biofilms by decomposing their extracellular matrices^29^ (for example, peroxides commonly used in denture cleaning)^9^ or act on the surface of a biofilm to disperse it^30^ (for example, the use of sodium lauryl sulfate or other surfactants). On the other hand, the saturated fatty acid salt did not remove the biofilm (Fig. 3d). Uncovering the key features responsible for the observed differences is important in order to understand this phenomenon; in particular, understanding the mode of action is important when considering the applications of this technology, such as how to further enhance the removal effect or achieve the same effect using other approaches.

By using OCT to observe the biofilm-removal process and ToF–SIMS to analyze the site of action of the fatty acid salt, we were able to demonstrate for the first time the relationship between localization of the unsaturated fatty acid salt at the interface and the formation of gaps. We believe that gaps are generated by the inflow of water due to a reduction in the interfacial energy caused by the presence of the fatty acid salt. The removal of a biofilm by common procedures involving peroxide or sodium lauryl sulfate, which progressively remove biofilms starting from their surfaces, require a long time to work because of their very nature. However, biofilm removal by an unsaturated fatty acid salt, as documented in this study, can be achieved in a very short time because it operates in a completely different manner, i.e., through detachment as a film. OCT also provided an important insight: the abovementioned gaps at the biofilm/PMMA-substrate interface are generated concurrently, independently, and irrespective of location, which suggests that the observed action is not a gradual process that proceeds in a stepwise manner from the surface of the biofilm. In this regard, the microstructural characteristics of the biofilms observed by SEM (Fig. 6) are important. On the macroscale, biofilms are often viewed as aggregated lumps; however, they have mesh-like membrane structures on the microscale, with “mountains and valleys” that range across the film. The FIB-SEM image of a cross-section of the biofilm confirmed that the thinnest region, shown in Fig. 7, is approximately 500-nm thick. In other words, unsaturated fatty acid salts only have to travel a short distance to reach the biofilm/PMMA-substrate interface.

Comparing data for various types of fatty acid salts that exhibit different effects is also important for understanding the nature of their actions. Site-of-action analysis by ToF–SIMS revealed that the fatty acid salts that cause the biofilm to detach were more concentrated at the interface than in the bulk, whereas laurate, which detached the biofilm, was less concentrated at the interface than in the bulk, which suggests that fatty acid localization at the interface contributes to biofilm removal. Furthermore, this result is consistent with the gap-generation behavior at the biofilm/PMMA-substrate interface observed by OCT. In other words, the action of a fatty acid salt at the biofilm/PMMA-substrate interface creates gaps at the interface, which causes the film to detach.

**Figure 8:**
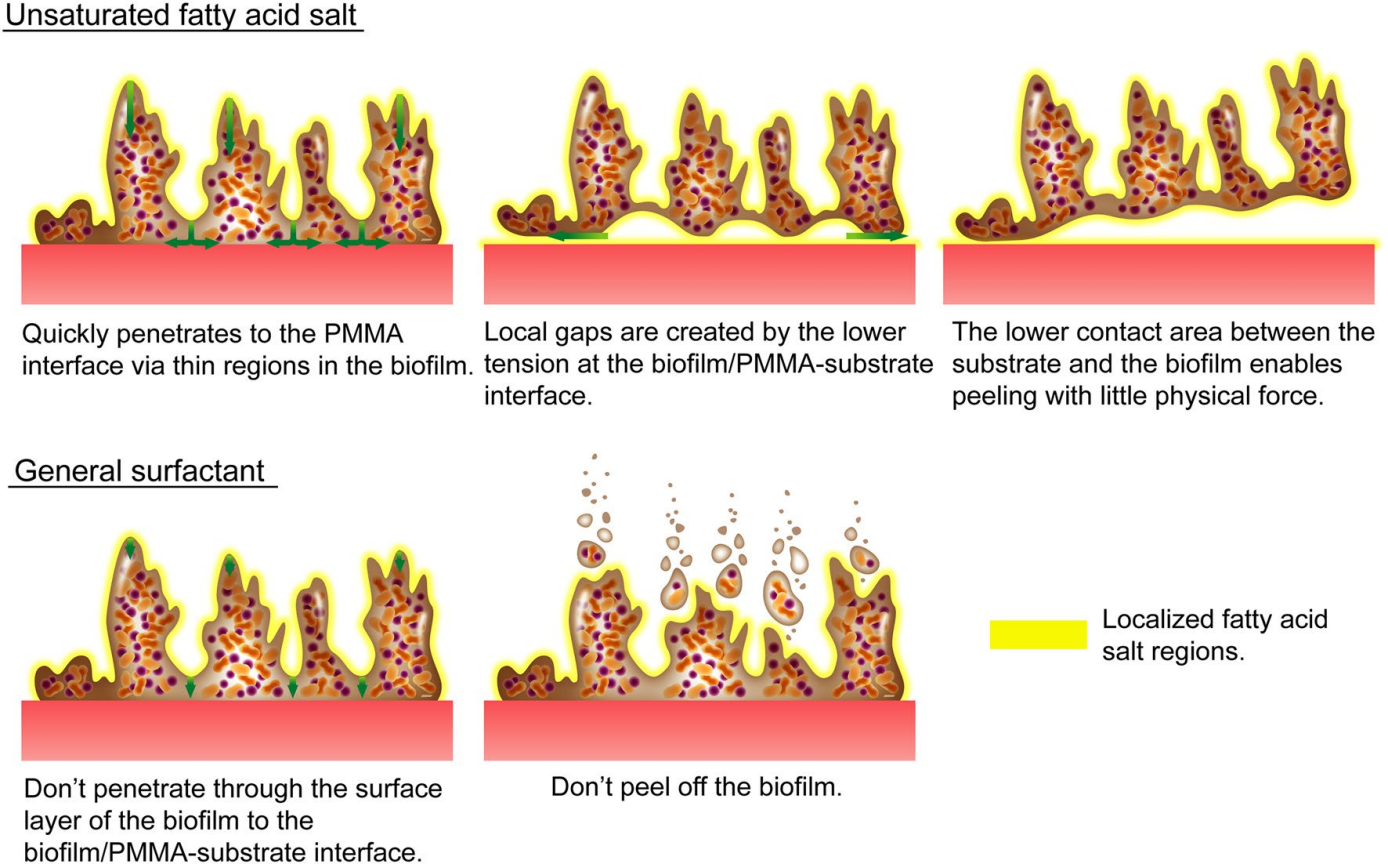
Biofilm-removal behavior of unsaturated and saturated fatty acid salts. Adobe Illustrator (CS) have been used to prepare this figure.

On the basis of the above findings, we proposed the action model as shown in Fig. 8. The unsaturated fatty acid salt quickly penetrates the surface layer of the biofilm through the thin areas of its mesh-like film to collect at the biofilm/PMMA-substrate interface. The unsaturated fatty acid salt acts as a surfactant that lowers the interfacial tension and causes mechanical distortions, such as osmosis-induced swelling, resulting in the formation of localized gaps, as observed by OCT. The PMMA/biofilm adhesion area decreases, which enables even the slightest physical force to detach the biofilm. In contrast, the saturated fatty acid salt cannot penetrate through the surface layer of the biofilm to the biofilm/PMMA-substrate interface, and hence, it is unable to detach the biofilm.

Understanding how unsaturated fatty acid salts can quickly penetrate to the interface to act on the biofilm remains a challenge for future work. Our hypothesis for explaining the difference in the behavior of unsaturated and saturated fatty acids is as follows: At room temperature, unsaturated fatty acid salts are in liquid state whereas saturated fatty acid salts are in solid state. It is speculated that the diffusion rate within the biofilm is significantly different for saturated and unsaturated fatty acid salts because of their different states. However, we anticipate that future studies on the interface and experimental and computational analyses of its affinity for biofilm matrices will provide an understanding of the mode of action at the molecular level.

In this study, we correlated the macroscopic observations of the biofilms with their microstructures to elucidate the important elements underlying their removal phenomena. This depth of understanding cannot be obtained using single analysis techniques only, and it provides an example of how biofilm research can benefit from a combination of multiscale analysis techniques and research approaches based on information integration.

Traditionally, brushing has been an effective means of removing biofilms on dentures^31,32^, but the effectiveness of brushing is skill-dependent^33,34^ and brushing coverage can be intermittent due to the complex shapes of dentures^35,36^. Existing denture cleaners that contain peroxides among other ingredients have certain ability to remove biofilms on their own, but their effectiveness is enhanced when combined with brushing^37^. In contrast, the removal of a biofilm from a denture surface using the unsaturated fatty acid salts documented in this study represents a significant improvement because treatment can be performed in a short time (approximately 10 min) and does not require brushing or other physical forces. The ability to remove biofilms simply and effectively from dentures in this manner not only makes it easier for the elderly to clean their dentures but also contributes to reducing diseases caused by oral biofilms.

## Conclusion

In this study, we demonstrated that unsaturated fatty acid salts are highly effective at removing biofilms from denture surfaces in a short time (approximately 10 min) using a method that does not require brushing or other physical forces. Oleate and linoleate, in particular, were highly effective in removing biofilms; treatment with these unsaturated fatty acid salts resulted in the detachment of biofilms from the surfaces of PMMA substrates. This phenomenon appears to be specific to unsaturated fatty acid salts and was not observed during treatment with a saturated fatty acid salt. We revealed that the ability of an unsaturated fatty acid salt to quickly penetrate the biofilm and create gaps at the biofilm/PMMA-substrate interface is important for achieving this specificity.

## Methods

### Materials

**Figure 9:**
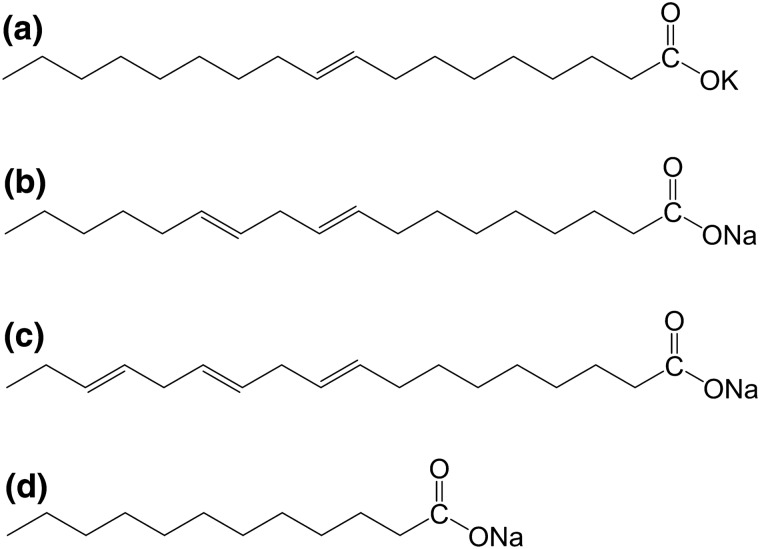
Structural formulas of fatty acid salts used in this study: (**a**) potassium oleate, (**b**) sodium linoleate, (**c**) sodium linolenate, and (**d**) sodium laurate.

Sodium oleate, potassium oleate, sodium laurate, and sodium linoleate (shown in Fig. 9) were obtained from TCI (Tokyo, Japan). Linolenic acid, sodium carbonate, sodium bicarbonate (anhydrous), 8 M sodium hydroxide solution, D( +)-glucose, sucrose, and calcium chloride (anhydrous) were purchased from the FUJIFILM Wako Pure Chemical Corporation (Osaka, Japan). We obtained DAIGO soybean-casein digest broth from the NIHON Pharmaceutical Co., Ltd. (Tokyo, Japan) and Bacto yeast extract from BD Japan (Tokyo, Japan). Crystal violet, 99.5% ethanol, and glutaraldehyde solution were purchased from Sigma-Aldrich (St. Louis, MO, USA), while PBS solution was obtained from the Nippon Gene Co., Ltd. (Tokyo, Japan). Polymethyl methacrylate (PMMA) prepared by the KYOYUKAI Co., Ltd. (Tokyo, Japan) was cut into 1 cm × 1 cm × 2 mm pieces and used as the substrate.

### Preparing the test solution

Sodium oleate, potassium oleate, sodium laurate, sodium linoleate, and linolenic acid were dissolved in deionized water to a concentration of 30 mM. The pH was adjusted to 10.3 by blending the solution with 0.22 wt% sodium carbonate and 0.17% sodium bicarbonate, followed by the addition of 8 M sodium hydroxide solution.

### In vitro biofilm formation

The following strains were obtained from the Gene Engineering Division of the RIKEN BioResource Research Center (Tsukuba, Japan): *Candida albicans* JCM1542 (hereinafter, *C. albicans*) and *Streptococcus mutans* JCM5705 (hereinafter, *S. mutans*). A liquid medium containing 3% DAIGO soybean-casein digest broth and 0.5% Bacto yeast extract (hereafter, SCD-YE medium) and a medium containing 1% D( +)-glucose and 2% sucrose added to the SCD-YE medium (hereinafter, the SCD-YE-GS medium) were prepared.

*C. albicans* was seeded in Anaero Columbia blood agar medium (Becton Dickinson, Tokyo, Japan) and incubated under aerobic conditions at 37 °C for 48–72 h. The colonies were diluted with SCD-YE medium as described earlier and incubated for 24 h at 37 °C under aerobic conditions, after which an SCD-YE medium was added to prepare a suspension with an optical density (OD) of 0.05. Then, *S. mutans* was seeded in an Anaero Columbia blood agar medium (Becton Dickinson, Tokyo, Japan) and incubated under anaerobic conditions at 37 °C for 48–72 h. Each colony was diluted in an SCD-YE medium and incubated under anaerobic conditions for 24 h at 37 °C. After incubation, the SCD-YE medium was added to prepare a bacterial suspension with an OD of 0.5.

The biofilm was formed by treating the PMMA substrate with a mixture of the *C. albicans* and *S. mutans* suspensions, as previously reported^38,39^. In addition, sodium chloride was added to the medium until it reached a concentration of 40 mg/L^40^. Specifically, the SCD-YE-GS medium, *C. albicans* suspension, *S. mutans* suspension, and sterile filtered 4.85% calcium chloride solution were mixed in a ratio of 1367:30:100:3 (v/v/v/v), respectively. The PMMA substrate was placed in each well of a 24-well plate (AGC Techno Glass Co., Ltd., Shizuoka, Japan), and 1 mL of the mixed medium was added, which was then incubated under anaerobic conditions for 16 h at 37 °C to yield a model biofilm substrate.

### In vitro biofilm-removal testing

The medium was removed from each well containing a biofilm model substrate, after which the well was rinsed with deionized water, and 1 mL of the relevant test solution was added. Each sample was left to stand at room temperature for 10 min and then shaken with 1 mL of water at 800 rpm for 5 min. This procedure was repeated three times. The samples were exposed to 0.75 mL of 0.1 wt% crystal violet solution for 15 min and then rinsed twice with water. The solution was diluted tenfold (vol.) with water after extracting the crystal violet with ethanol, and the residual amount of biofilm was evaluated by absorbance (OD 595 nm) using a TECAN plate reader (FUJIFILM Wako Pure Chemical Corporation, Tokyo, Japan). The biofilm-removal rate was determined using the following formula:

$${\text{Biofilm}}\;{\text{removal}}\;{\text{rate}}\left( \% \right) = \frac{{A_{c} - A_{b} - \left( {A_{s} - A_{b} } \right)}}{{\left( {A_{c} - A_{b} } \right)}} \times 100,$$

where A_c_, A_b_, and A_s_ are the absorbances of the control, blank, and sample, respectively.

### Microstructural observations (SEM)

SEM images were acquired using an FE-SEM S-4800 microscope (Hitachi High-Tech Corporation, Tokyo, Japan) at an accelerator voltage of 5 kV.

After testing the removal, 1 mL of a 2.5% aqueous glutaraldehyde solution was added to each biofilm model substrate and allowed to stand for 1 h. The glutaraldehyde solution was then removed, and the substrates were washed with PBS and immersed in 50%, 70%, 90%, and 99.5% ethanol/PBS solutions for 3 min each before being dried with a blower.

For SEM, each PMMA substrate was fixed on an aluminum cylinder stub (EMJapan Co., Ltd., Tokyo, Japan) using carbon tape (Okenshoji Co., Ltd., Tokyo, Japan) and then conductively treated using an osmium coater (HPC-30 W: Vacuum Device Co., Ltd., Ibaragi, Japan).

### Cross-sectional biofilm structure (FIB-SEM)

Cross-sectional structures were observed using a FIB-SEM system (Scios DualBeam FEI, Tokyo, Japan). The same biofilm model substrates used for SEM were cut with a gallium ion beam (30 kV, 100 pA, and 15 nA) over an area (100 × 50 × 30 µm) large enough to include both the thick and thin regions of the microbiota before being observed at an accelerator voltage of 5 kV.

### Observing biofilm detachment (OCT)

OCT was performed using full-field OCT (FF-OCT, LLTech Inc., Paris, France). The images were acquired in the planar direction over an 800 µm × 800 µm area to depths between 250 and 300 µm.

The PMMA substrate on which the biofilm had formed was fixed to the measurement cell using carbon tape (Okenshoji Co., Ltd, Tokyo, Japan) on an aluminum stub, and 40 µL of deionized water was dripped onto the biofilm surface. The height was adjusted so that the top surface of the biofilm was not in contact with the glass surface of the cell, after which the OCT procedure was commenced. The number of scans per image was set to 250. Tilt correction, 3D reconstruction and area fraction analysis were performed using image analysis software (Fiji and FluoRender).

### Fatty acid salt site-of-action analysis (ToF–SIMS)

ToF–SIMS was performed using a PHI TRIFT V nanoTOF instrument (ULVAC-PHI, Inc., Chigasaki, Japan). This system is equipped with a primary ion gun for measurement purposes and an ion gun for sputtering purposes. The incident angle of both ion sources is 40° relative to the normal direction of the sample surface. The accelerator voltage was set to 30 kV, with Bi_3_^2+^ as the primary ion. ToF–SIMS data were acquired in high mass-resolution mode (m/Δm = 9000; lateral resolution = 0.5 μm) with a raster scan over a 100 × 100 μm area. An Ar_2500_^+^ argon gas cluster ion (GCIB) with an average cluster size of approximately 2,500 was used as the ion gun for sputtering during depth profiling. GCIB sputtering was performed by raster scanning over a 500 × 500 μm area at an accelerator voltage of 15 kV and an average current of approximately 5 nA.

The biofilm samples that had detached from the PMMA substrates were freeze-dried and attached to the sample holder using double-sided adhesive and conductive carbon tape, followed by depth profiling of each base material. The primary Bi_3_^2+^ ion fluence was configured to be lower than the static limit (1 × 1012 ions/cm^2^) in each experiment. A dual-beam neutralizer with an electron gun and argon ion gun was used for charge compensation. The GCIB irradiation time was set to 60 s during depth profiling (the sputtering rate was estimated to be approximately 2 μm/min). Measurements and sputtering were repeated for 20 cycles. The CH_3_^+^, C_2_H_3_^+^, and C_3_H_5_^+^ peaks were used for positive ions, and the CH^−^, C_2_H^−^, and OH^−^ peaks were used for negative ions to mass-calibrate the ToF–SIMS spectra. To represent the depth profile, the ion intensity of each fatty acid was normalized against the ion intensity of protein-derived CNO^−^ (*m/z* 42), and the maximum intensity of each profile was set to unity.

### Statistical analysis

Data are presented as means ± s.e.m. One-way analysis of variance (ANOVA) with a Tukey’s honestly significant difference (HSD) test was performed for multi-group comparison testing, with p < 0.05 considered to be statistically significant. IBM SPSS Statistics version 22 (IBM, New York, USA) statistical analysis software was used.

## Supplementary Information

Supplementary Information.
