# Supplementary material for: Unsaturated fatty acid salts remove biofilms on dentures
Source: Sci Rep. 2021 Jun 15;11:12524. doi: 10.1038/s41598-021-92044-y (PMC8206114; doi:10.1038/s41598-021-92044-y)
Supplement: Supplementary file 1 — Supplementary Information. [file 41598_2021_92044_MOESM1_ESM.pdf]

**Supporting information**

# Unsaturated Fatty Acid Salts Remove Biofilms on Dentures

Teruyuki Hara[a†], Atsunori Sonoi[b†], Takuya Handa[b], Masayuki Okamoto[a], Eri Kaneko[a], Reiko Ikeda[a], Taichi Habe[a], Hidetake Fujinaka[b], Shigeto Inoue[a*], Tetsuo Ichikawa[c*]

[a]Analytical Science Research Laboratories, Kao Corporation, 1334 Minato, Wakayama-shi, Wakayama 640-8580, Japan

[b]Personal Health Care Products Research Laboratories, Kao Corporation, 2-1-3, Bunka, Sumida-ku, Tokyo 131-8501, Japan

[c]Department of Prosthodontics and Oral Rehabilitation, Graduate School of Biomedical Sciences, Tokushima University, Tokushima 770-8504, Japan

[†]These authors contributed equally

[*]Corresponding authors: inoue.shigeto@kao.com, ichi@tokushima-u.ac.jp

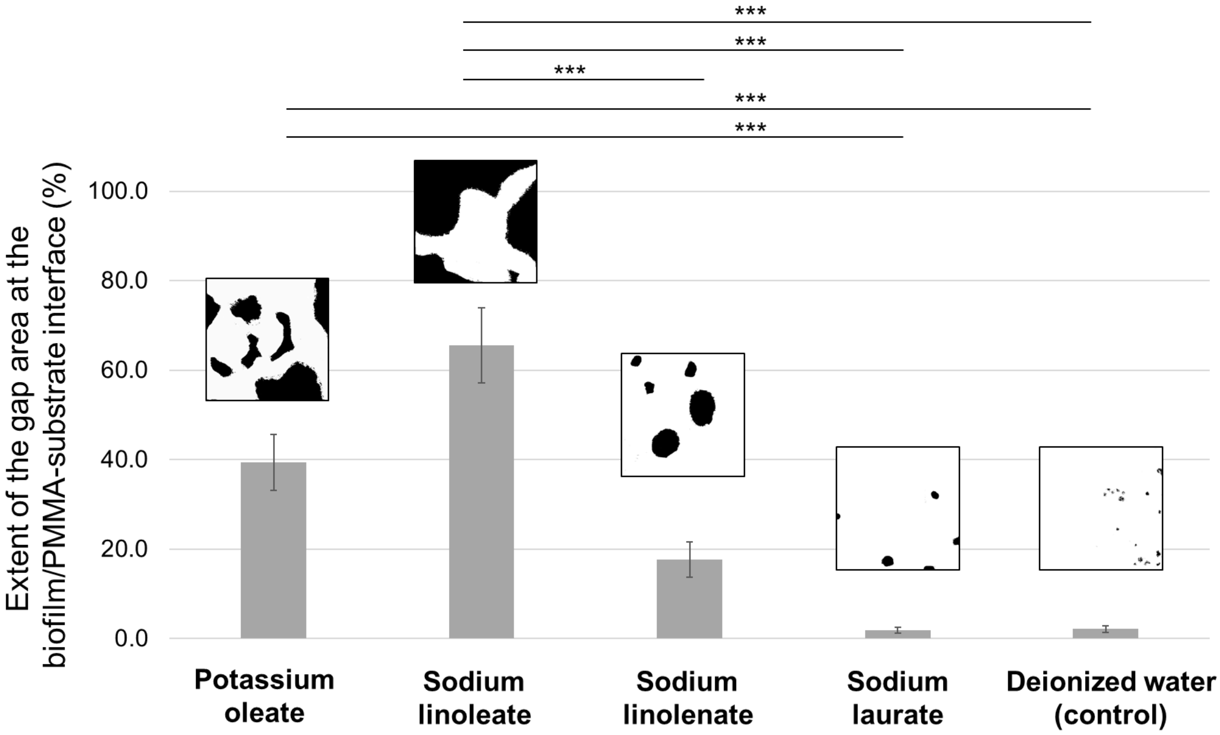


**Figure S1.** Extent of gap area at the biofilm/PMMA-substrate interface obtained from optical coherence tomography (OCT) images of co-cultured *C. albicans* and *S. mutans* biofilms after immersion in deionized water and various 30 mM fatty acid salt solutions for 10 min. (a) Oleate, (b) linoleate, (c) linolenate, (d) laurate and (e) deionized water. Typical x-y cross-sectional images at biofilm/PMMA-substrate are shown inset (800 µm × 800 µm). The biofilms are depicted in white and gaps in black. The results are shown as means ± s.e.m. (n = 5). ***: $p<0.005$ for the comparison among groups. The asterisks indicate statistically significant differences between the groups.
